# Autophagy-associated alpha-arrestin signaling is required for conidiogenous cell development in *Magnaporthe oryzae*

**DOI:** 10.1038/srep30963

**Published:** 2016-08-08

**Authors:** Bo Dong, Xiaojin Xu, Guoqing Chen, Dandan Zhang, Mingzhi Tang, Fei Xu, Xiaohong Liu, Hua Wang, Bo Zhou

**Affiliations:** 1 State Key Laboratory of Breeding Base for Zhejiang Sustainable Pest and Disease Control, Institute of Virology and Biotechnology, Zhejiang Academy of Agricultural Sciences, Hangzhou 310021, Zhejiang Province, China; 2 College of Chemistry and Life Science, Zhejiang Normal University, Jinhua 321104, Zhejiang Province, China; 3 State Key Laboratory of Rice Biology, China National Rice Research Institute, Hangzhou 310006, Zhejiang Province, China; 4 State Key Laboratory of Rice Biology and Key Laboratory of Chinese Ministry of Agriculture for Nuclear-Agricultural Sciences, Zhejiang University, Hangzhou 310029, China; 5 Institute of Digital Agriculture, Zhejiang Academy of Agricultural Sciences, Hangzhou 310021, Zhejiang Province, China; 6 State Key Laboratory of Rice Biology, Biotechnology Institute, Zhejiang University, Hangzhou 310058, Zhejiang Province, China; 7 Institute of Crop and Nuclear Technology Utilization, Zhejiang Academy of Agricultural Sciences, Hangzhou 310021, Zhejiang Province, China; 8 International Rice Research Institute, DAPO Box 7777, Metro Manila 1301, Philippines

## Abstract

Conidiation patterning is evolutionarily complex and mechanism concerning conidiogenous cell differentiation remains largely unknown. *Magnaporthe oryzae* conidiates in a sympodial way and uses its conidia to infect host and disseminate blast disease. Arrestins are multifunctional proteins that modulate receptor down-regulation and scaffold components of intracellular trafficking routes. We here report an alpha-arrestin that regulates patterns of conidiation and contributes to pathogenicity in *M. oryzae*. We show that disruption of *ARRDC1* generates mutants which produce conidia in an acropetal array and *ARRDC1* significantly affects expression profile of *CCA1*, a virulence-related transcription factor required for conidiogenous cell differentiation. Although germ tubes normally develop appressoria, penetration peg formation is dramatically impaired and Δ*arrdc1* mutants are mostly nonpathogenic. Fluorescent analysis indicates that EGFP-ARRDC1 puncta are well colocalized with DsRed2-Atg8, and this distribution profile could not be altered in Δ*atg9* mutants, suggesting ARRDC1 enters into autophagic flux before autophagosome maturation. We propose that *M. oryzae* employs ARRDC1 to regulate specific receptors in response to conidiation-related signals for conidiogenous cell differentiation and utilize autophagosomes for desensitization of conidiogenous receptor, which transmits extracellular signal to the downstream elements of transcription factors. Our investigation extends novel significance of autophagy-associated alpha-arrestin signaling to fungal parasites.

Conidia are asexual spores used by quite many fungal pathogens for infection initiation and disease dissemination. At least twenty modes of conidiation have been described in conidial fungi^1^, including the causal agent of the rice blast disease *Magnaporthe oryzae*^2^,^3^, which generates conidia in a sympodial pattern^4^.

Foliar blast disease starts with aerial dispersal of sympodial conidia through wind and raindrop splash^5^. Conidia attach to host cuticles and produce germ tubes that differentiate into appressoria with huge turgor pressure^6^. Appressoria develop penetration peg to breach host surfaces allowing subsequent infection hyphae to ramify throughout surrounding epidermis cells^7^. Four to five days later, conidiophores emerge from infected sites and sympodial conidia are produced for secondary dissemination^4^,^8^.

Upon favorable environmental cues, the rice blast fungus differentiates conidiogenous cell at the tip of aerial conidiophore stalk. Mono-nucleus conidiogenous cell subsequently undergoes mitotic divisions to give a three-celled conidium. Then the active fertile site on the conidiophore stalk moves sideways to produce a second conidium. Several rounds of conidiation ultimately generate multiple sympodial conidia on a mature conidiophore^4^,^9^. In spite of such a clear mycological characterization of conidiogenous cell development in *M. oryzae*, very little is currently known regarding mechanism modulating spore patterning and its contribution to pathogenicity except for two transcription factors, *ACR1* and *CCA1*^9^,^10^. *ACR1* encodes a transcription factor homologous to *MEDA* of *Aspergillus nidulans* and *REN1* of *Fusarium oxysporum*, both of which been characterized as developmental regulators for correct conidial differentiation^11^,^12^,^13^. Δ*acr1* mutants produce conidia in an acropetal array, impair in appressorium formation and are nonpathogenic^9^. *CCA1* was identified from a systematical analysis of 104 fungal-specific Zn_2_Cys_6_ transcription factors in *M. oryzae*. Δ*cca1* mutants sporulate in a way similar to that in Δ*acr1* mutants and could not bring about foliar blast disease because of defects in conidial germination and appressorium formation^10^. It was proposed that diverse modes of conidiation in conidial fungi arose through alteration in major genes controlling spore-specific gene expression^9^.

Arrestins are adaptor and scaffold proteins that assemble molecules involved in signaling events such as signal transduction and intracellular protein trafficking, gathering associated proteins within spatial proximity and thus facilitating their proper interaction^14^,^15^. This adaptor/scaffold function was initially characterized in the mammalian visual rhodopsin and beta-adrenergic system^16^,^17^, and the proteins were termed as visual/beta-arrestins for their capacity to bind to the activated G-protein-coupled receptors, preclude ligand-receptor interaction and arrest subsequent signaling^18^. It is now known that beta-arrestins also function in modulating other cell surface proteins such as growth factor receptors and ion-transport proteins^19^,^20^. In addition to the plasma membrane receptors, accumulating data show that beta-arrestins interact with a burgeoning list of cytosolic targets, for instances, component of mitogen-activated protein kinase cascades and regulatory proteins such as the tumor suppressor p53^15^,^21^, thereby involved in regulating a wide variety of cellular processes^22^.

Beyond the well-studied mammalian visual/beta-arrestins, a much wider and ancient family of arrestin proteins has been recently discovered in organisms from protists to mammals except plants, and referred to as alpha-arrestins^23^,^24^. Alpha-arrestins and visual/beta-arrestins lack sequence similarity between each other, but share the same tertiary structure of N- and/or C-terminal arrestin-domain^25^,^26^, and possess analogous function in trafficking steps of membrane-embedded proteins. However, as the more ancient branch that emerged relatively recently^24^, alpha-arrestins may simply function in a wider diversity of signaling routes than do visual/beta-arrestins^22^.

Members of mammalian alpha-arrestins include arrestin-domain containing protein 1–5 (ARRDC1-5) and TXNIP (Thioredoxin-interacting protein)^25^. Because of their recent discovery, the detail function of ARRDCs is less known and increasing data show that ARRDCs play various roles in regulation of membrane proteins and prominently function in the mediation of metabolism^22^,^27^. Alpha-arrestins in *Saccharomyces cerevisiae* or arrestin-related trafficking adaptors (ARTs) bind to cell surface receptors in response to stresses such as pH and heavy metals, and function by distinct mechanisms to internalize plasma membrane cargoes via either clathrin-mediated or clathrin-independent endocytosis^28^,^29^,^30^,^31^,^32^. Additionally, ARTs can negatively regulate yeast pheromone response via internalization and desensitization of the G-protein-coupled receptors Ste2^33^.

PalF in *A. nidulans* is one of the filamentous fungal alpha-arrestins whose role is best understood^34^,^35^,^36^,^37^,^38^,^39^, which constitutes a critical component of the conserved ambient pH-signaling pathway involved in fungal pathogenicity^40^,^41^,^42^,^43^. PalF is activated in a signal and receptor-dependent pattern similar to that in the mammalian beta-arrestins, and activated PalF binds the pH receptor PalH and recruits the component of ESCRT compartments to the cortical pH signaling sites, which stimulates further process that mediates fungal adaptation to ambient pH. Lately, a systematic investigation of alpha-arrestins in *A. nidulans* has been documented in respect to growth, morphology, drug sensitivity and specifically turnover of the uric acid-xanthine transporter UapA. This study, in combination with a previously reported alpha-arrestin CreD, puts the basis for understanding critical roles of alpha-arrestins in down-regulation of cell surface receptors in the filamentous fungi^44^,^45^. Except for that in *A. nidulans*, characterization of alpha-arrestins other than PalF is barely documented in the remaining filamentous fungi.

Macroautophagy (herein referred as autophagy) is a highly conserved self-degradative pathway that regulates numerous physiological and pathological processes in eukaryotic cells^46^. The morphological hallmark of autophagy is incorporation of intracellular proteins and organelles into a double-membrane sequestering compartment termed the phagophore. The phagophore subsequently expands and matures into an autophagosome which eventually transports to and fuses with vacuoles/lysosomes for cargo degradation and recycling^47^. A subset of autophagy-related genes (*ATG*s) has been discovered that gathers at the pre-autophagosomal structures or phagophore assembly site (PAS), and cooperates with the forming phagophore to generate a mature autophagosome^48^,^49^. Upon vesicle completion, most of these ATG proteins are dismissed except for Atg8. Some Atg8 stays association with the completed autophagosome and enters into the vacuolar lumen, making Atg8 an ideal marker that tracks the autophagy flux^50^.

Here we describe characterization of an alpha-arrestin required for correct differentiation of conidiogenous cells in the filamentous fungus *M. oryzae*. We show that Δ*arrdc1* mutants conidiate in an acropetal way and dramatically reduce in pathogenicity. Fluorescent analysis strongly suggests that ARRDC1 is recruited into nascent autophagosomes during their biogenesis before maturation. We propose that ARRDC1 acts as an adaptor protein that internalizes its cargoes such as conidiogenous receptors on fertile sites of the conidiophore stalk, and assembles autophagosomes for endocytosis of conidiogenous receptor, which transmits conidiogenous signals to the downstream transcription factors. The identification of ARRDC1 unveils alpha-arrestins as a novel class of genes involved in a mycological morphogenesis insufficient of documentation and our discovery extends significance of autophagy-associated alpha-arrestin signaling to microbial pathogenicity.

## Results

### ARRDC1 putatively encodes is an arrestinC-domain containing protein

Using a BlastP analysis with arrestinN (pfam00339) and/or arrestinC (pfam02752) against the *M. oryzae* genome, we have identified seven arrestin-domain containing proteins referred to as ARRDC1-6 and MoPalF. The domain organization within proteins is schematically represented in Figure S1.

Because fungal alpha-arrestins have only been investigated in the yeast and *Aspergillus* species, we compared the rice blast fungal alpha-arrestins with that in *S. cerevisiae* and *A. nidulans*, respectively. In comparison with a relatively lower similarity between homologues in *M. oryzae* and *S. cerevisiae* (Table S1), alpha-arrestins are relatively well conserved in the two filamentous fungi (Table S2).

Here, we chose to focus on ARRDC1 because deletion of *ARRDC1* generates mutants with striking defects not so often documented in the filamentous fungus (see below).

### Construction of Δarrdc1 mutants

To investigate the cellular function of *ARRDC1*, we performed one-step gene replacement strategy (Figure S2A). The gene disruption vector was introduced into rice pathogenic strain KJ201, and hygromycin-resistant transformants were selected from two independent fungal transformation performances. DNA gel blot analysis identified six gene replacement mutants, of which three are shown (Figure S2B). All six replacement mutants display the same defective phenotypes, and one null mutant was used for phenotypic analysis and complementation assay.

### ARRDC1 deletion causes defects in conidiogenous cell development

**Figure 1:**
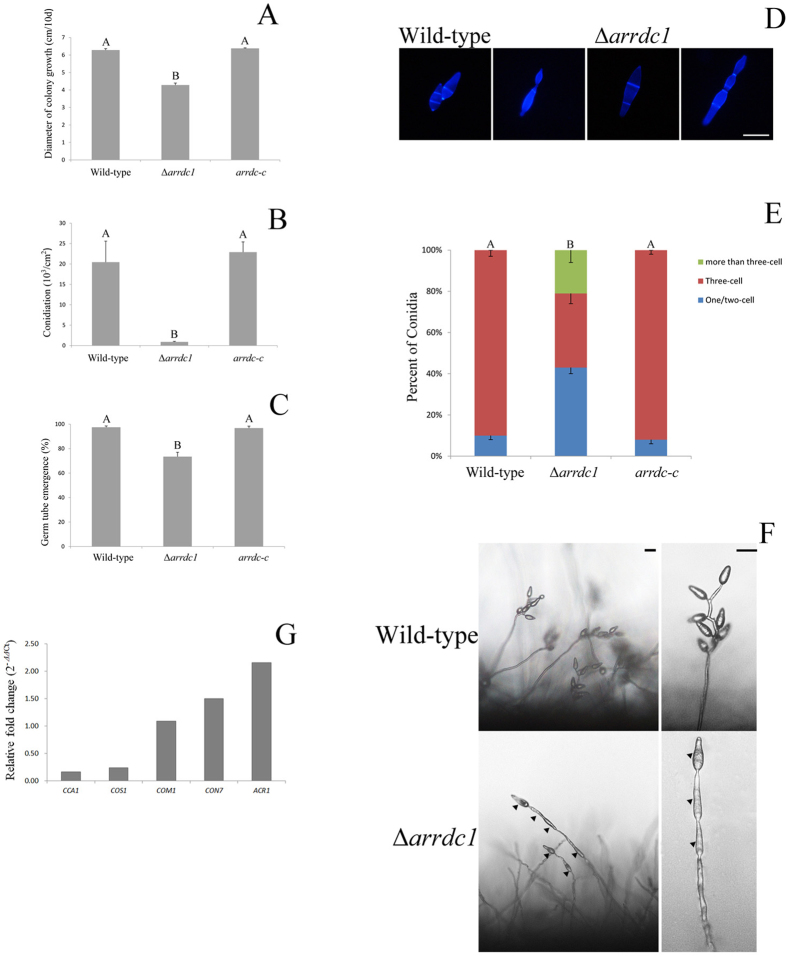
Conidiogenous cell development of *M. oryzae* strains. (**A**) Colony growth of *M. oryzae* strains on CM agar plates. The wild-type strain, Δ*arrdc1* and complementation strain *arrdc-c* were cultured at 25 °C for 10 days. The same capital letters on top of columns indicate non-significant differences as estimated by Duncan’s test (P ≤ 0.05). (**B**) Conidiation of *M. oryzae* strains. The strains were cultured at 25 °C for 10 days, and then conidiation recorded. The same capital letters on top of columns indicate non-significant differences as estimated by Duncan’s test (P ≤ 0.05). (**C**) The conidial germination rate of *M. oryzae* strains. Conidia were dropped on plastic coverslips and incubated at 25 °C for 48 h. The same capital letters on top of columns indicate non-significant differences as estimated by Duncan’s test (P ≤ 0.05). (**D**) The conidial morphology of the wild-type and Δ*arrdc1* mutants. Conidia from each strain were collected and stained with Calcofluor White. Bar = 20 μm. (**E**) Statistical analysis of conidia with numbers of septa. The Δ*arrdc1* mutants produce conidia with various numbers of septa. More than 300 conidia were counted for each strain. The same capital letters on top of columns indicate non-significant differences as estimated by Duncan’s test (P ≤ 0.05). (**F**) Conidiophore development of the wild-type and Δ*arrdc1* mutants. Δ*arrdc1* mutants gave less conidiophore and generated conidia in an acropetal way, and the wild-type produced conidiated in a sympodial pattern. Arrows indicate conidia produced in a head-to-tail way in the Δ*arrdc1* mutants. Bar = 20 μm. (**G**) Transcriptional expression patterns of conidiation-related transcription factors in the Δ*arrdc1* mutants. Relative expressions of *CON7*, *COS1*, *COM1*, *ACR1* and *CCA1* were measured in the Δ*arrdc1* mutants during conidiogenous cell development. Transcription levels were normalized to the endogenous gene beta-tubulin and relative values calculated by fold change (2^−*ΔΔ*Ct^) compared to each transcript in conidiogenous cell development of the wild-type. Total RNA was prepared from conidiogenous mycelia of the wild-type and Δ*arrdc1* mutants.

We investigated the role of *ARRDC1* in colony growth and sporulation. We found that Δ*arrdc1* mutants grew slower than the wild-type KJ201 (Fig. 1A). Conidiation in Δ*arrdc1* mutants was dramatically reduced, and mutants typically produced 20-fold fewer spores compared with the wild-type (Fig. 1B). We then tested conidial germination by calculating germ tube emergence between the wild-type and mutant strains, and found that 97.50 ± 1.04% of the wild-type conidia germinated within 2 h of exposure to water, while only 73 ± 3.45% of mutant conidia retained the ability to germinate even after a prolonged inoculation of up to 48 h (Fig. 1C). And the wild-type typically produced uniform conidia with two septa that define three cells in a conidium, but conidia produced by Δ*arrdc1* mutants constantly enlarged and contained septa of variable numbers, as stained with Calcofluor White to visualize with cell walls and septa (Fig. 1D,E).

We then examined the process of sporulation in KJ201 and Δ*arrdc1* mutants. Aerial hyphae were erased with sterile distilled water, mycelia blocks cut and placed under constant illumination with high humidity for 24 h, and conidial development was observed. We found that the wild-type produced conidia in a sympodial pattern, in which multiple conidia emerge at several fertile sites on a single conidiophore stalk. A next round of conidial formation originates at the conidiophore stalk following termination of a previous round of conidiation. In contrast, conidiophore formation was reduced in the Δ*arrdc1* mutants and conidia were produced in an acropetal way. Although aerial conidiophore stalks undergo terminal swelling to produce the first conidium, a second conidium always shows up at the tip of the first one, and subsequently a third conidium emerges at the tip of the second conidium. Ultimately several conidia are linked in a chain and anchored on one fertile site of the conidiophore stalk (Fig. 1F). We conclude that the wild-type conidiates in a sympodial way and Δ*arrdc1* mutants produce conidia in an acropetal pattern.

### Gene expression profile of CCA1 is affected in Δarrdc1 mutants

Many more pathogenicity-related transcription factors have been identified to be required for conidiation^51^, however, scarcity of investigation is involved in genes related to spore patterning except for *ACR1* and *CCA1*. We thus investigated the expression profiles of *ACR1* and *CCA1* in Δ*arrdc1* mutants during conidiation. Other transcription factors we tested include *CON7*, *COS1* and *COM1*. Absence of *CON7* and *COM1* results in abnormal conidial morphology^52^,^53^, and *COS1* is required for conidiophore formation^54^. We found that *CCA1* (0.16-fold) and *COS1* (0.24-fold) were significantly down-regulated in the conidiogenous mycelia of Δ*arrdc1*, as compared with the conidiogenous mycelia of the wild-type (Fig. 1G).

### ARRDC1 is necessary for pathogenicity

**Figure 2:**
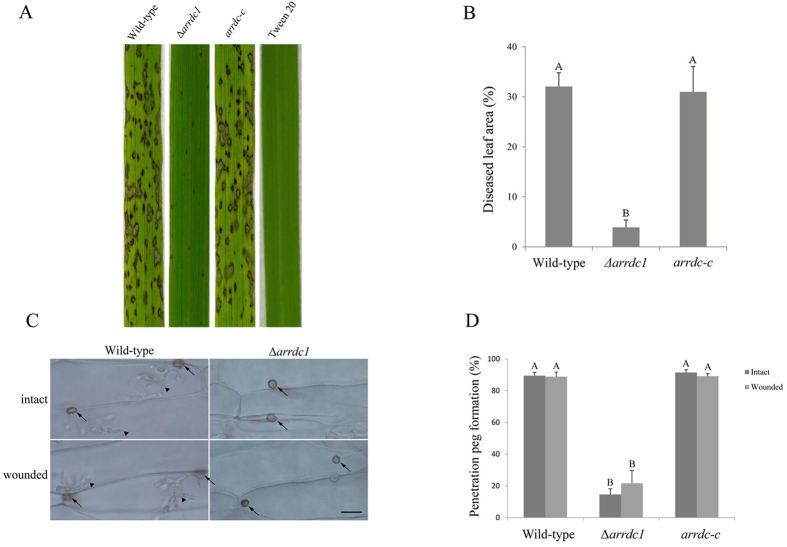
Pathogenicity of Δ*arrdc1* mutants. (**A**) Susceptible rice seedlings were sprayed with conidia suspensions (5 × 10^4^/ml) of the wild-type, Δ*arrdc1* mutants and the complementation strains from 12-day-old CM plates. Photographs were taken at 5-day post-inoculation. (**B**) Data are presented as a bar chart showing percentage of lesion areas. The same capital letters on top of columns indicate non-significant differences as estimated by Duncan’s test (P ≤ 0.05). (**C**) Penetration peg formation and invasive growth were observed with intact and wounded barley leaves. 30 μl of conidial suspensions (5 × 10^4^/ml) were inoculated on leaf explants. Photographs were recorded at 30 h after incubation in high humidity chamber. The triangle indicates invasive hyphae and the arrow indicates appressoria. Bar = 20 μm. (**D**) Statistical analysis of penetration peg formation. The same capital letters on top of columns indicate non-significant differences as estimated by Duncan’s test (P ≤ 0.05).

To determine the role of *ARRDC1* in pathogenesis, we inoculated two-week-old seedlings of a rice cultivar CO-39 with conidial suspensions of Δ*arrdc1* mutants and the wild-type. A representative result of four spraying assays is shown in Fig. 2A,B. While host of the wild-type displayed densely distributed leaf symptoms, dot lesions sparsely occurred on plants inoculated with Δ*arrdc1* mutants.

### ARRDC1 is dispensable for appressorium formation and turgor generation

We performed a detailed examination of virulence-associated function to explore the reason why Δ*arrdc1* mutants dramatically reduce in pathogenicity. *In vitro* assays of appressorium formation showed that germ tubes in Δ*arrdc1* mutants formed appressoria similar to that of the wild-type (not shown). We then asked whether *ARRDC1* is required for full turgor generation in the appressorium. Foliar blast infection in the rice blast fungus is a turgor-driven process associated with substantial glycerol accumulation in the appressoria^55^. We performed incipient cytorrhysis assays in which the rate of appressorium collapse is determined in the hyperosmotic concentration of glycerol^56^. We found no significant differences in appressorial turgor between Δ*arrdc1* mutants (76.00 ± 3.77% collapsed appressoria in the presence of 2 M glycerol) and the wild-type (77.72 ± 2.01% collapsed appressoria at 2 M glycerol).

### ARRDC1 is required for penetration peg formation

Because barley is a natural host for *M. oryzae*, we used barley leaves to investigate penetration peg formation between Δ*arrdc1* mutants and the wild-type. Epidermal penetration assays were carried out to allow appressoria to form on both intact barley leaves and those in which the cuticles were removed firstly by abrasion. Formation of penetration peg was calculated 30 h after inoculation. We found that 14.60 ± 3.61% appressoria of null mutants produced penetration hyphae compared with 89.47 ± 2.01% in the wild-type appressoria. And a similar result was observed on abraded leaves (Fig. 2C,D). Thus, ARRDC1 is dispensable for appressorium formation and turgor generation, but required for penetration peg emergence.

### Reintroduction of ARRDC1 restores colony growth, patterns of conidiation, penetration peg formation and pathogenicity

We performed a complementation assay to ask whether the phenotypes were attributable to *ARRDC1* disruption. Complementation analysis was performed via introducing a 4.5-kb genomic fragment spanning the *ARRDC1* gene into the null mutant. Transformants were selected, and the presence of a single copy of *ARRDC1* was confirmed by DNA gel blot analysis (data not shown). Seedlings sprayed with conidia from transformants carrying the 4.5-kb fragment exhibited normal rice blast disease symptoms. And colony growth, sporulation, patterns of conidiation and penetration peg formation were all restored by complementation performance (Figs 1 and 2).

### EGFP-ARRDC1 resides on different diameters of punctate sites

To elucidate the intracellular distribution of ARRDC1, we initially constructed four fusion proteins of EGFP tagged to both ends of ARRDC1 under control of its endogenous promoter and the H3 promoter to transform the Δ*arrdc1* mutants^57^. Both gene fusions were able to complement the defects of colony growth, spore patterning and pathogenicity in the Δ*arrdc1* mutants (data not shown). But sufficient signals could only be detected with gene fusions under the H3 promoter. Because EGFP tagged to both ends of ARRDC1 displays similar patterns of cellular distribution and EGFP-ARRDC1 gives more observable fluorescent signal, we thus used transformants expressing EGFP-ARRDC1 to perform confocal microscopy analysis.

**Figure 3:**
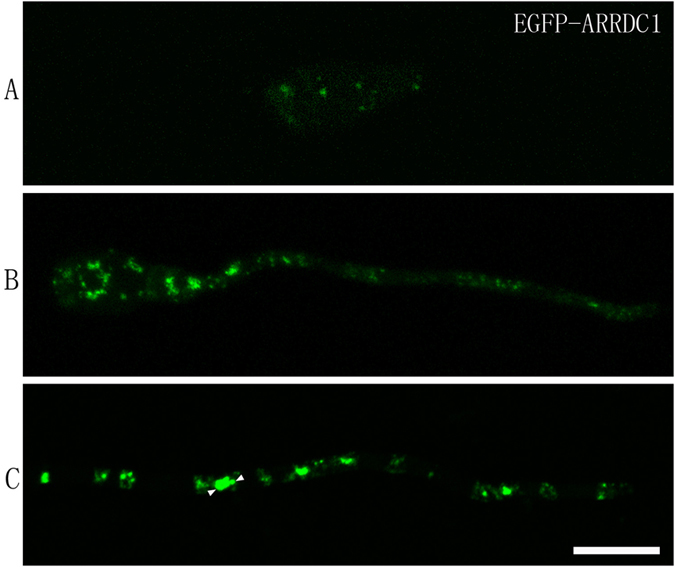
EGFP-ARRDC1 distributes on different diameters of punctate sites. (**A**) In nascent conidia, multiple dim spots of EGFP-ARRDC1 among the entire cytoplasm were constantly captured. (**B**) EGFP-ARRDC1 signals become strong and plenty of distinct punctate sites were observed in the germinating conidium and its germ tube upon 8 h inoculation. (**C**) EGFP-ARRDC1 fluorescent dots vary considerably in diameters in the vegetative hypha developed from conidia prolonging inoculation time up to 24 h, and some dots become very large and are connected with smaller dots as indicated by the white triangles. Bar = 10 μm.

In mycelia plugs collected from transformants on solid plates, we constantly captured multiple small dots of dim fluorescence distributed throughout the cytoplasm of the nascent conidia, and observed intensive punctate signals in the hyphae. We asked whether EGFP-ARRDC1 resides on different types of organelles or small dots undergo homotypic fusion to generate large-sized structures. We then inoculated conidia with CM media on microscope coverslips and carefully observed EGFP-ARRDC1 fluorescent signals during conidial development into vegetative hypha. In the dormant spore, EGFP-ARRDC1 was constantly captured to be distributed on several dim spots (Fig. 3A). After 8 h inoculation, fluorescent signal became strong and plenty of distinct punctate sites showed up in germinating conidia and germ tubes (Fig. 3B). Prolonging inoculation time up to 24 h, we found that EGFP-ARRDC1 fluorescence varied considerably in diameters and some dots became very large and were connected with smaller dots (Fig. 3C). We conclude that EGFP-ARRDC1 is distributed on multiple punctate vesicles of different diameters.

### EGFP-ARRDC1 puncta are well colocalized with DsRed2-Atg8

The endocytic route is a major pathway responsible for recognition and transport of cargoes targeted to the vacuole and the Rab7-positive late endosome is a crucial intermediate during endocytosis in most cell types. In *M. oryzae*, Ramanujam, Naqvi and colleagues discovered that the late endosomal compartments, apart from membrane trafficking, regulate the biogenesis and spatio-temporal dynamics of cAMP signaling essential for pathogenesis through anchoring active G-protein signaling components^58^. Considering that alpha-arrestins possibly act as critical adaptors for turnover of cell surface proteins, we initially carried out colocalization assay between EGFP-ARRDC1 and MCHERRY-Rab7, but no overlapping was observed between the two fluorescent signaling (Figure S3).

**Figure 4:**
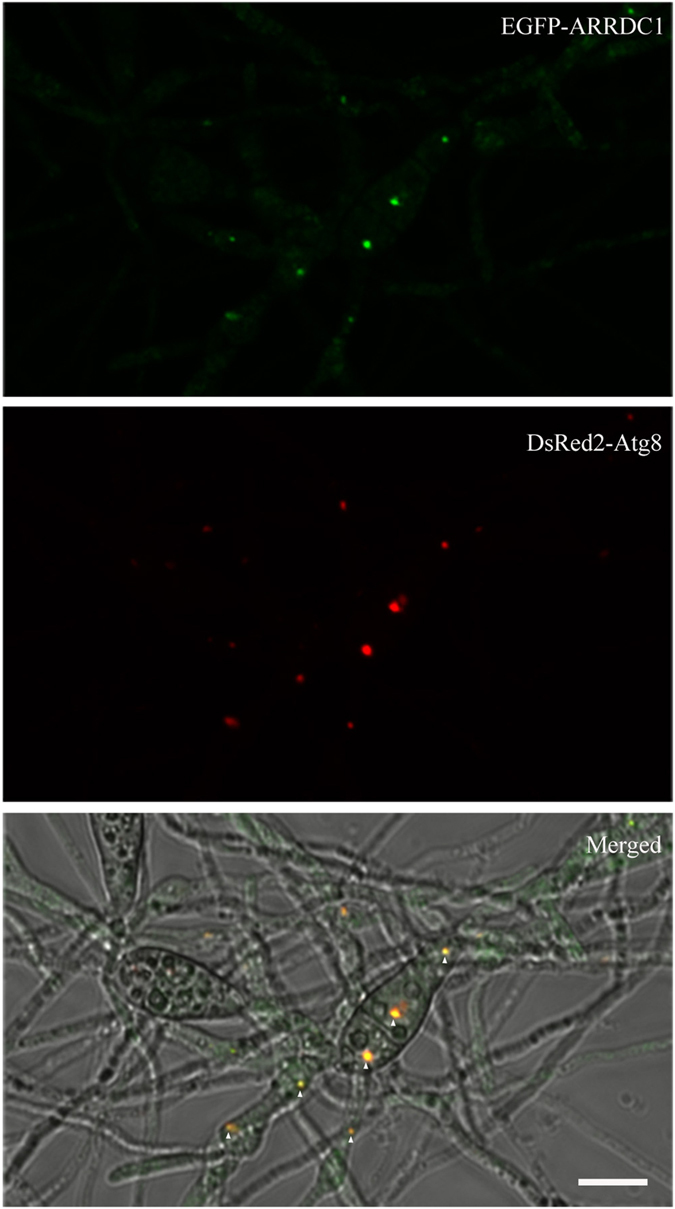
EGFP-ARRDC1 is colocalized with DsRed2-Atg8. Conidia coexpressed with EGFP-ARRDC1 and DsRed2-Atg8 were collected and inoculated on plastic coverslips with nutrient media allowing development of vegetative hypha. The white triangles indicate different diameters of colocalized sites between EGFP-ARRDC1 and DsRed2-Atg8 in the condia and vegetative hypha. Bar = 10 μm.

Our previous study showed that multiple PAS sites in *M. oryzae*, indicated by colocalization of EGFP-Atg9 and DsRed2-Atg8, distributed on different diameters of puncta signals which constantly undergo homotypic fusion into larger structures^59^. Because the distribution pattern of EGFP-ARRDC1 bears a strong resemblance to the localization profile of PAS sites, we subsequently performed a colocalization analysis between EGFP-ARRDC1 and DsRed2-Atg8. We found that puncta of EGFP-ARRDC1 were well colocalized with DsRed2-Atg8, leaving other green signals distributing throughout the entire cytoplasm (Fig. 4) (79% of n = 106 ARRDC1 puncta colocalized with Atg8 dots).

**Figure 5:**
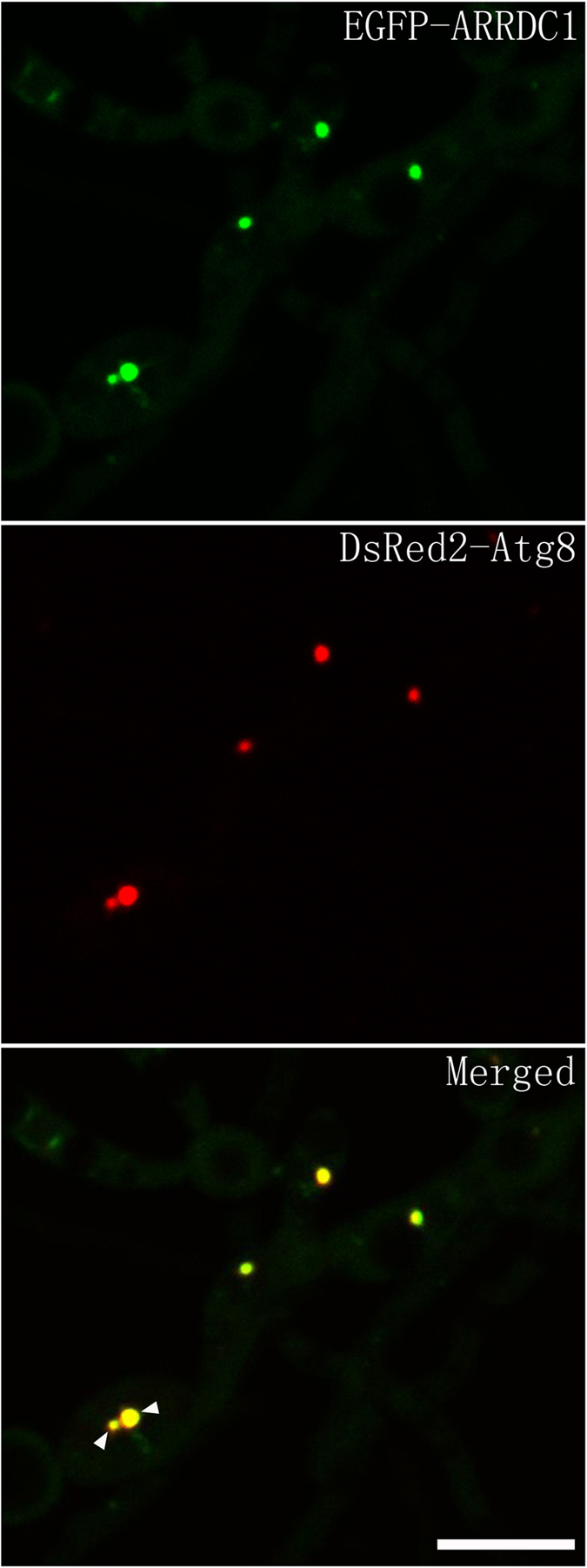
Colocalization of EGFP-ARRDC1 with DsRed2-Atg8 in the autophagy-defective Δ*atg9* mutants. EGFP-ARRDC1 is colocalized with DsRed2-Atg8 in the absence of autophagy process. The white arrows indicate two closely colocalized sites in a single cell of vegetative hypha. Bar = 10 μm.

To further assess colocalization of EGFP-ARRDC1 with autophagic compartments. We generated Δ*atg9* mutants in the background of the wild-type stain KJ201 via targeted gene replacement using the deletion vector we previously constructed^59^. Δ*atg9* mutants in the background of KJ201 display similar defects as that in the background of another wild-type strain GUY11 as we previously reported (data not shown). We then co-expressed EGFP-ARRDC1 and DsRed2-Atg8 in Δ*atg9* mutants, and found that EGFP-ARRDC1 and DsRed2-Atg8 were still well colocalized with each other (Fig. 5) (75% of n = 100 ARRDC1 puncta colocalized with Atg8 dots). Because Δ*atg9* mutants are defective in autophagosome formation and introduction of ARRDC1 and/or Atg8 could not complement the autophagic defects in the Δ*atg9* mutants. We conclude that EGFP-ARRDC1 puncta is colocalized with the PAS structures and autophagosomes.

## Discussion

In the current study, we have provided a novel documentation concerning alpha-arrestin of a fungal pathogen in virulence-associated cell differentiation, especially in conidiogenous cell development and penetration peg formation. As a first endeavor towards understanding alpha-arrestin pathway in the rice blast fungal pathogenicity, we have identified seven alpha-arrestins in the genome of *M. oryzae*. While deletion of arrestins such as *ARRDC2* or *ARRDC3* gives no obvious effect (not shown), possibly because of their functional redundancy, absence of *ARRDC1* leads to pleiotropic defects underlining the indispensable significance of this alpha-arrestin. The most striking phenotype of Δ*arrdc1* mutants is its inability to establish a normal sympodial spore patterning and the mutants conidiate in an acropetal chain, i.e., a second conidium forms at the tip of a former one instead of a nearby fertile site on conidiophore stalks. Although germ tubes generate appressoria with normal turgor pressure, penetration peg development and invasive hypha growth are impaired and the mutants exhibit dramatically reduced disease symptoms.

Conidiogenous cell development is evolutionarily complex and characterization of genes concerning spore patterning is insufficiently documented and mainly concentrated on transcription factors^1^,^9^,^12^,^13^,^60^. In *M. oryzae*, only two transcription factors, *CCA1* and *ACR1*, have been identified that are required for correct establishment of sympodial conidiation. And Δ*cca1* and Δ*acr1* mutants all produced conidia in an acropetal way similar to that observed in the Δ*arrdc1* mutants^9^,^10^. Because appressorium formation failed in Δ*cca1* and Δ*acr1* mutants, it was reasonably suggested that appressorium differentiation was impaired because of aberrant pattern of conidiogenous cell development^9^. In contrast, we found that although loss of *ARRDC1* produced conidia with reduced germination rate, germ tubes normally gave appressoria comparable to the wild-type in respect of appressorium formation and turgor generation. We reason that *CCA1* and *ACR1* may function in multiple pathways including conidiation and appressorium formation, and our results imply that conidiogenous cell development and subsequent appressorium differentiation are not two intrinsically dependent happenings as suggested before.

Quite many phytopathogenic fungi, including *M. oryzae*, employ conidia as infectious propagules for disease initiation and dissemination. And conidia are produced in at least twenty patterns with mechanisms rarely known^1^. One way fertile hypha tips check status of vegetative growth and initiate conidiation is through remodeling plasma membrane proteins responsible for conidiogenous cell differenation. Because alpha-arrestins serve as critical regulators for turnover of cell surface proteins^28^,^45^,^61^, we suggest that upon favorable environmental cues, specific cell surface receptors transport to the fertile tip of conidiophore stalks to initiate conidiogenous cell development, and then recycle back to a second fertile site for next round of conidiation. Ultimately, *M. oryzae* produces several conidia directly linked to its center conidiophore stalk in a sympodial pattern, so that every nascent conidium is well nutritionally accommodated. Under natural conditions, *ARRDC1* strictly modulates the conidiogenous receptor and prevents it from repeatedly catalyzing signaling activation of conidiation. In contrast, deletion of *ARRDC1* disorganizes internalization and recycling of the conidiogenous receptor in the mutants, and the conidiogenous receptor is not promptly removed from fertile sites, prolonging signals energizing for conidiation. Thus, the next conidium generates from the tip of a former conidium, instead of another fertile tip in conidiophore stalks. We propose that *M. oryzae* employs alpha-arrestins like *ARRDC1* to control specific conidiogenous receptors at fertile tips of conidiophore stalk and senses suitable stimulus to accurately initiate and terminate conidiation, possibly through controlling expression of downstream transcription factors such as *CCA1*, and the conidiogenous receptors have yet to be identified.

Autophagy is a strictly regulated process and autophagy activity is primarily modulated by number and size of autophagosomes. While the number of autophagosomes indicates the frequency of autophagosome formation, alteration in the diameters of autophagosomes allows sequestration of different quantities and dimensions of cargoes^62^. In the budding yeast cell, one PAS is regularly observed, in contrast with multiple PAS sites in the mammalian cell. Our previous study showed that in *M. oryzae*, multiple small Atg8-positive sites are easily detected in a single cell, and these structures constantly undergo homotypical fusion into large Atg8-positive structures^59^, resembling the homotypic fusion of two LC3-positive vesicles or Atg16L-positive autophagosome precursors in the mammalian cell^63^,^64^. Ultimately, these larger dimensions of Atg8-positive structures appear to merge into a center site, possible in concomitant with the rice blast fungal morphogenesis of small spherical vacuoles developing into a central vacuole. Here, we found that EGFP-ARRDC1 clearly colocalizes with DsRed2-Atg8 puncta and undergoes the same pattern of subcellular trafficking as that of Atg8-positive structures. And this pattern of colocalization is not altered in the Δ*atg9* mutants in which formation of autophagosomes is defective and DsRed2-Atg8 represents the PAS structures. We reason that ARRDC1 is not just transiently colocalized with autophagosomes, it enters into autophagic flux at the cargo recognition stage before maturation of phagophore into autophagosome.

Except for MVBs in the endocytic pathway, autophagosomes in the selective autophagy process emerge as alternative organelles responsible for recognition and transport of cargoes targeted to the vacuole^65^,^66^. These specific cargoes expand from entire organelles to signal molecules. For instances, the mammalian Notch signaling, a master regulator crucial for stem cell differentiation, is modulated by autophagy via uptake of the plasma membrane receptor Notch1 into ATG16L1-positive autophagosome precursor vesicles for degradation^67^. And by promoting degradation of Dishevelled, autophagy negatively regulates Wnt signaling which performs key functions in cell development, tissue self-renewal and tumorigenesis^68^. The *Arabidopsis thalinana* membrane-anchored TSPO is a multi-stress regulator that is transiently induced by abiotic stresses and its accumulation is strictly down-regulated through the autophagic pathway^69^,^70^. Here, we further propose that ARRDC1 acts as an adaptor/scaffolding protein that internalizes its cargoes like conidiogenous receptors on the fertile sites of the conidiophore stalk as we suggested above, and recruits autophagy-related vesicles for subsequent turnover of receptor proteins. Reciprocally, this autophagy-associated alpha-arrestin signaling route may provide a large pool of membrane materials and regulators on the plasma membrane for autophagosome formation without compromising other membrane trafficking events.

The identification of *ARRDC1* unveils alpha-arrestins as an important catalog of genes involved in fungal parasites during their infection process. Further investigation of alpha-arrestins in the rice blast pathogen and other fungal parasites will auspiciously extend potential significance of alpha-arrestin mediated pathway to fungal pathogenicity and broaden insight into the molecular basis of microbial virulence.

## Experimental Procedures

### Fungal strains and growth conditions

*M. oryzae* strains were cultured on complete medium (CM) plates and preserved on filter paper disks as previously described^71^,^72^. KJ201 was used as the wild-type and all mutants mentioned in this study were generated from KJ201. CM plates were incubated at 25 °C under 12 h light/dark cycles.

### Targeted gene replacement and fungal transformation

Construction of the replacement vector was performed using double-joint PCR as previously described^73^. Basically, two flanking sequences of the *ARRDC1* locus were amplified from the genomic DNA of the *M. oryzae* wild-type KJ201 with primers 3241u1/u2 and 3241d1/d2, respectively. A 1.4 kb hygromycin B phosphotranferase gene (*HPH*) cassette was cloned from pCB1003 with primers Hphm1/m2. The three amplicons were linked together and served as the template for the final double-joint PCR amplified with nested primers 3241n1/n2. The double-joint PCR product was then inserted into the *EcoR*I/*BamH*I sites of pCAMBIA1300 to obtain the final gene replacement vector. The replacement vector was introduced into *Agrobacterium tumefaciens* strain AGL1 and *Agrobacterium-tumefaciens* mediated transformation (ATMT) was performed as previously described^74^. Resistant transformants were selected on CM plates supplemented with 250 μg/mL hygromycin B for gene replacement and 800 μg/mL G418 for complementation assay, respectively. The multiplex PCR with primers 3241ck1/ck2 and Hphck1/ck2 was used for the first round of mutant screening. The candidate mutants were purified from single conidium and southern blot analysis was performed to confirm the homology recombination and single integration event. For complementation assays, a 4.5-kb full-length copy of the *ARRDC1* locus including its native promoter and terminator region was amplified from the genomic DNA using primers 3241c1/c2, and inserted into a modified pCAMBIA1300 vector containing a geneticin resistance gene. The resulting construct was randomly inserted into the genome of Δ*arrdc1* mutants via ATMT and southern blot analysis was carried out to verify successful single-copy integration. All primers used for gene replacement and complementation analysis are listed in Table S3.

### Southern blot analysis

Genomic DNA was extracted by standard CTAB protocols. Agarose gel separation, restriction enzyme digestion and Southern hybridization analysis were performed following the standard procedure and the protocol provided within the digoxigenin (DIG) high prime DNA labelling and detection starter Kit I (Roche, Germany). For Southern blot, genomic DNA was digested with *Sca*I and labeled with the probe amplified using primers 3241sb1/sb2. The primers are listed in Table S3.

### Phenotypic analysis

To visualize the conidiophore formation and pattern of conidiation, aerial hyphae from 5-day cultures growing on 90 mm CM plates were erased with sterile distilled water, and then mycelia blocks were cut and placed under constant illumination with 90% humidity for 24 h.

For sporulation test, strains were grown on three independent CM plates. After 10-day of growth, the spores were harvested and counted. To stain septa, appropriately diluted conidia (1 × 10^5^/ml) collected from CM agar plates, were incubated on glass films in a moist chamber at 25 °C, samples were soaked with Calcofluor White in the dark for 2 min before epifluorescence microscopy examination.

Appressorial development assays were performed on hydrophobic microscope coverslips (Fisherbrand). Average values were determined from at least 200 spores and performed in triplicate. Appressorial turgor was estimated by incipient cytorrhysis assays^55^. Briefly, conidia of 20 μl droplets (5 × 10^5^/ml) were incubated on microscope coverslips in a humid chamber for 24 h and induced to form appressoria. Water embracing appressoria was removed carefully and replaced with 20 μl of glycerol in concentration of 2 M. The number of appressoria that had collapsed after 5 min was recorded. Average values were determined from at least 200 spores and performed in triplicate.

Penetration peg formation was determined as previously described^75^. Simply, conidia were collected and resuspended to the concentration of 5 × 10^4^/ml with 0.02% (wt/vol) Tween 20. A 20 μl droplet was deposited onto the upper side of leaves cut from 8-day-old barley (Hordeum vulgare cv. ZJ-8), and maintained on 4% (wt/vol) water agar plates at 25 °C for 30 h. The leaves that showed disease lesions were decolored by methanol, stained in lactophenol with 0.01% trypan blue-0.06% aniline blue, and observed under a microscope for host penetration. Images were taken using an Eclipse 80i microscope (Nikon).

### Pathogenicity assays

Fourteen-day-old rice seedlings of the susceptible dwarf indica cultivar, CO-39, were infected with suspensions of *M. oryzae* conidia prepared in 0.02% tween 20, at a concentration of 5 × 10^4^/ml, using Badger airbrush. Plants were incubated in plastic bags for 36 h to maintain high humidity and then transferred to controlled environmental chamber at 22 °C and 90% relative humidity for 72 h for full disease symptoms to develop. The infected leaves were collected at 5-day post-inoculation and images taken using an Epson Workforce scanner at a resolution of 2400 dpi.

### Plasmid construction of pEGFP-ARRDC1, pMCHERRY-Rab7 and pDsRed2-Atg8

Fragment of *ARRDC1* was amplified with primers arrdc1-xba1/2 from mycelia cDNA of the wild-type strain KJ201, and inserted into the *Xba*I site of pKD5GFP to generate the pEGFP-ARRDC1. The pMCherry-Rab7 was constructed via insertion of Rab7 cDNA amplified with rab7-xba1/2 into the *Xba*1 site of pKD3cmcherry, and the H3 promoter was replaced with the RP27 promoter amplified with primers rp27-1/2. Note that Rab7 expressed under the H3 promoter is mislocalized and signals diffusely distributed among the entire cytoplasm. All ligation reactions were performed with Infusion Kit from Clontech. The pKD5GFP and pKD3mcherry plasmids are kind gifts from Dr. Jianping Lu at College of Life Sciences, Zhejiang University^57^. pDsRed2-Atg8 was constructed according to our previous description^59^. Resistant transformants were selected on CM plates, supplemented with 800 μg/ml G418 for expression of pDsRed2-Atg8 and 500 μg/ml glufosinate ammonium for expression of pMCherry-Rab7, respectively. Transformants expressed pEGFP-ARRDC1 were selected on DCM (yeast nitrogen base without amino acid 1.7 g/l, NH_4_NO_3_ 2 g/l, L-asparagine 1 g/l, glucose 10 g/l, and Na_2_HPO_4_ was used to adjust pH to 6.0) supplemented with 100 μg/mL chlorimuron-ethyl. The primers for fluorescent constructs are listed in Table S3.

### Quantitative Real-Time PCR assay

RNA was isolated with the Trizol reagent (Invitrogen) following the manufacturer’s recommendation. A 2000 ng of total RNA was added to synthesize the first strand cDNA using SYBR ExScriptTM RT-PCR kit (Takara). Real-time PCR reaction was performed with SYBR Premix Ex Taq (Takara) on a C1000 thermal cycler (Bio-rad). Relative abundance of transcripts was calculated using the 2^−*ΔΔ*Ct^ method with beta-tubulin (MGG00604) as the endogenous control. Thermocycler conditions were as follows: 3 min at 95 °C, followed by 45 cycles of 95 °C for 15 s, 60 °C for 15 s, and 72 °C for 15 s. A final dissociation cycle was incorporated to ensure the specificity of each primers. Data were collected from at least three independent experiments with three replicates, and one representative set of results was presented. Primers used for Real-Time PCR analysis are listed in Table S3.

## Additional Information

**How to cite this article**: Dong, B. *et al*. Autophagy-associated alpha-arrestin signaling is required for conidiogenous cell development in *Magnaporthe oryzae*. *Sci. Rep.* **6**, 30963; doi: 10.1038/srep30963 (2016).

## Supplementary Material

Supplementary Information
