# Corrigendum: Autophagy-associated alpha-arrestin signaling is required for conidiogenous cell development in *Magnaporthe oryzae*

**DOI:** 10.1038/srep33939

**Published:** 2016-09-30

**Authors:** Bo Dong, Xiaojin Xu, Guoqing Chen, Dandan Zhang, Mingzhi Tang, Fei Xu, Xiaohong Liu, Hua Wang, Bo Zhou

Scientific Reports 6: Article number: 3096310.1038/srep30963; published online: 08 08 2016; updated: 09 30 2016

This Article contains a typographical error in the Acknowledgements section.

“We would also like to thank Li Xe and Xijiao Song (Zhejiang Academy of Agricultural Sciences, Hangzhou, China) for technical assistance on the confocal microscopic analysis”.

should read:

“We would also like to thank Li Xie and Xijiao Song (Zhejiang Academy of Agricultural Sciences, Hangzhou, China) for technical assistance on the confocal microscopic analysis”.

