# Supplementary material for: Autophagy-associated alpha-arrestin signaling is required for conidiogenous cell development in Magnaporthe oryzae
Source: Sci Rep. 2016 Aug 8;6:30963. doi: 10.1038/srep30963 (PMC4976345; doi:10.1038/srep30963)
Supplement: Supplementary Information [file srep30963-s1.pdf]

Supplementary information

Autophagy-associated alpha-arrestin signaling is required for conidiogenous cell development in *Magnaporthe oryzae*

Bo Dong, Xiaojin Xu, Guoqing Chen, Dandan Zhang, Mingzhi Tang, Fei Xu, Xiaohong Liu, Hua Wang, Bo Zhou

Figure S1. Identification of arrestin-domain containing proteins in the rice blast fungus. The fold organization of the rice blast fungal arrestin-domain containing proteins is schematically represented. Seven alpha-arrestins are identified in the rice blast fungal genome referred to as ARRDC1-6 and MoPalF. ArrN and ArrC stands for arrestinN and arrestinC domain, respectively. LDB19 belongs to ArrN domain.

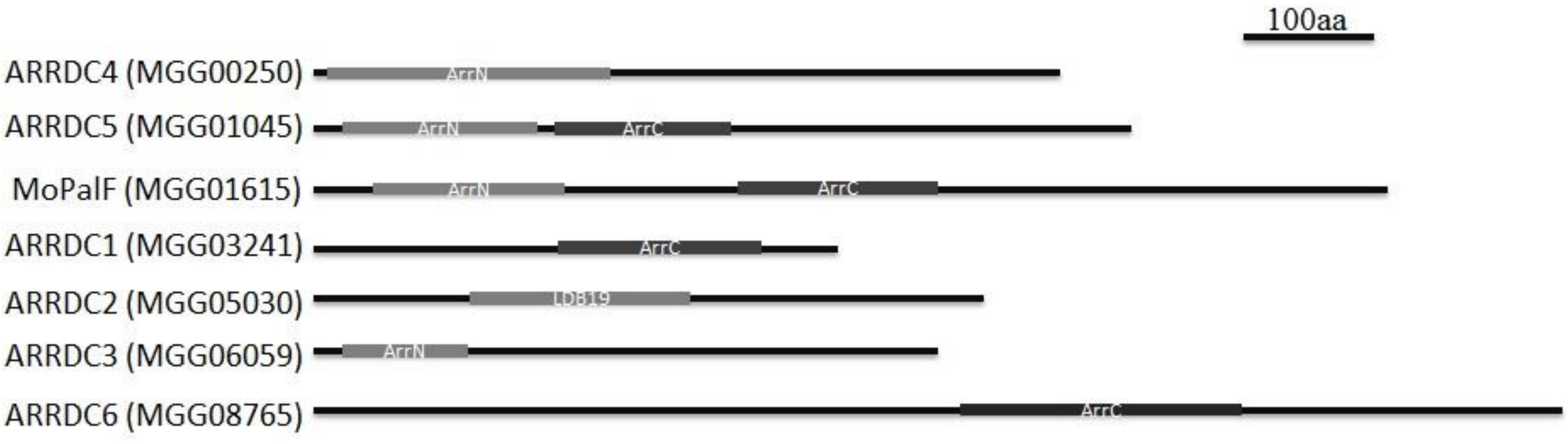

Figure S2. Generation of Δ*arrdc1* mutants. A. Targeted gene replacement of the *ARRDC1* gene. The gene knock-out vector pKOarr1 was used for transformation of the wild-type *M. oryzae* strain KJ201. B. Southern blot analysis of the wild-type strain KJ201 (lane 1) and Δ*arrdc1* strains (lane 2-4). Genomic DNA was digested with *Sca*I and hybridized with the probe. Sc, *Sca*I.

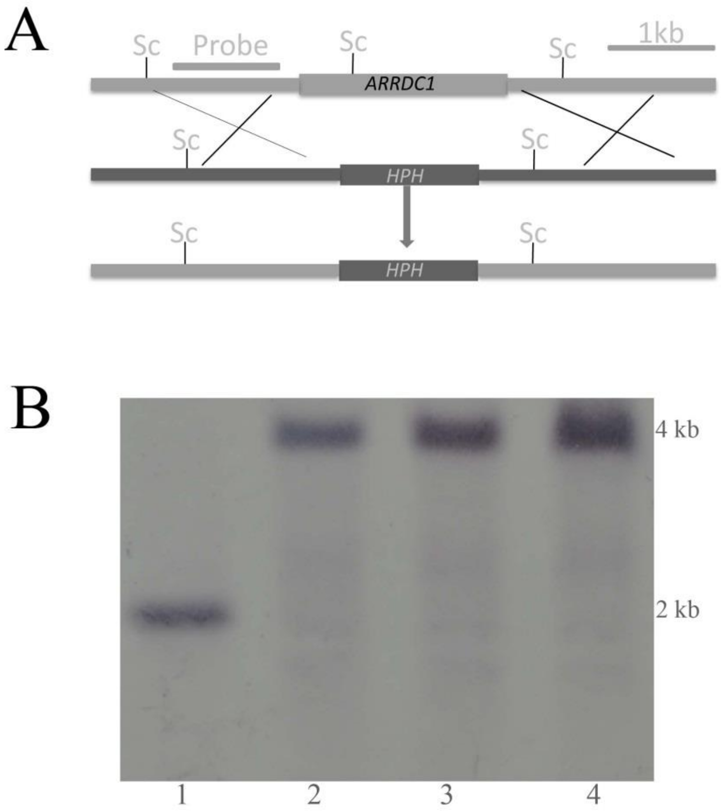

Figure S3. EGFP-ARRDC1 is not colocalized with MCHERRY-Rab7. Conidia co-expressed with EGFP-ARRDC1 and MCHERRY-Rab7 were harvested and inoculated on microscope coverslips for vegetative hypha development. EGFP-ARRDC1 is not overlapped with MCHERRY-Rab7. Bar=10μm.

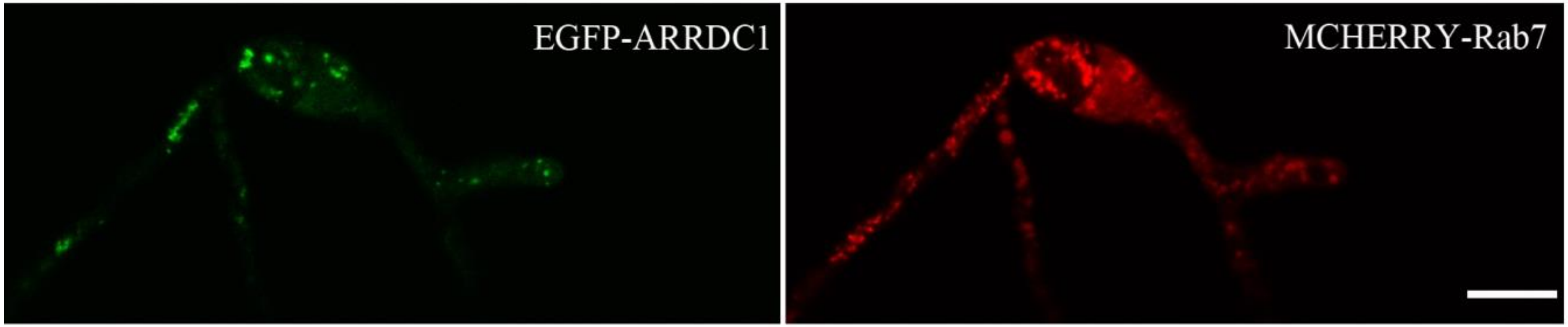

Table S1: Comparison of alpha-arrestins in *M. oryzae* and *S. cerevisiae* performed using the EMBOSS Needle online tool. The numbers without bracket indicate amino acids identity between *M. oryzae* and *S. cerevisiae*. Total score resulted from the Needle tool is shown in brackets. ARRDC1 and ADDRC3 show relatively lower similarity to yeast alpha-arrestins. ARRDC2, ARRDC4, ARRDC5, ARRDC6 and MoPalF are significantly more similar to Art1 (18.6% identity), Art10 (19.3% identity), Art4 (24.8% identity), Art6 (18.9% identity) and Art9 (17% identity) than to any other alpha-arrestins of *S. cerevisiae*, respectively.

	Art1 YOR322C 818aa	Art2 YBL101C 1117aa	Art3 YJL084C 1046aa	Art4 YOR018W 837aa	Art5 YGR068C 586aa	Art6 YKR021W 915aa	Art7 YFR022W 733aa	Art8 YPR030W 1121aa	Art9 YGL045W 542aa	Art10 YLR392C 518aa
ARRDC1 MGG03241 402aa	10.7 (56.5)	8.9 (77.5)	7.9 (72.5)	12 (57)	11.9 (31)	9.6 (79.5)	6.6 (40.5)	8.6 (47.5)	13.9 (50.5)	6.9 (32.5)
ARRDC2 MGG05030 514aa	**18.6 (391.5)**	10.2 (101)	9.9 (79)	9.9 (92)	18.6 (113)	10.3 (72)	11.4 (81.5)	10.1 (110)	14.8 (49)	15.7 (33.5)
ARRDC3 MGG06059 479aa	12.1 (49)	8.5 (82.5)	9.9 (55)	10.6 (62)	12.1 (69.5)	6.2 (61.5)	13.1 (71)	8.4 (71)	17 (48.5)	17.1 (56)
ARRDC4 MGG00250 572aa	14.8 (71)	5.3 (59)	11.2 (85.5)	15 (104)	13.1 (50.5)	11.3 (49.5)	15.6 (91.5)	11.2 (65.5)	17.4 (94)	**19.3 (176)**
ARRDC5 MGG01045 627aa	17.4 (116)	12.4 (113.5)	12.6 (137.5)	**24.8 (621.5)**	15.2 (262)	16.1 (175)	24.7 (589.5)	12 (103)	16 (95)	14.8 (68)
ARRDC6 MGG08765 958aa	14.6 (78.5)	18.4 (301.5)	17.6 (662.5)	13.7 (219)	13.7 (211)	**18.9 (676)**	15.9 (261.5)	174 (313)	12.5 (54.5)	9.9 (39)
MoPalF MGG01615 824aa	10.8 (90.5)	14.6 (142.5)	16.2 (102.5)	15.8 (132)	11.8 (49.5)	17.2 (117)	17.2 (127.5)	13.5 (112)	**17 (377)**	10.4 (76.5)

Table S2: Comparison of alpha-arrestins in *M. oryzae* and *A. nidulans* performed using the EMBOSS Needle online tool. The numbers without bracket indicate amino acids identity. Total score resulted from the Needle tool is shown in brackets. Alpha-arrestins are high conserved in *M. oryzae* and *A. nidulans*. Note that ARRDC1 shows the highest score and % amino acid identity to its *Aspergillus* orthologue among all the rice blast fungal alpha-arrestins.

	ArtA<br>ANID_00056.1<br>521aa	ArtB<br>ANID_1089.1<br>813aa	CreD<br>ANID_04170.1<br>597aa	ApyA<br>ANID_03265.1<br>368aa	PalF<br>ANID_01844.1<br>766aa	ArtC<br>ANID_01741.1<br>289aa	ArtD<br>ANID_09105.1<br>609aa	ArtE<br>ANID_02447.1<br>401aa	ArtF<br>ANID_03302.1<br>407aa	ArtG<br>ANID_05453.1<br>496aa
ARRDC1<br>MGG03241<br>402aa	12.8 (30.5)	10.5 (44.5)	7.8 (28.5)	6.4 (44)	11.2 (56.5)	13.1 (52.5)	5.5 (47.5)	**65.7 (1475)**	15.1 (43.5)	15.1 (42)
ARRDC2<br>MGG05030<br>514aa	**31.5 (712)**	15.8 (154.5)	12.9 (116)	15 (144)	12 (63)	13.1 (55.5)	12.1 (59)	8 (30.5)	6 (42.5)	17 (43.5)
ARRDC3<br>MGG06059<br>479aa	15.9 (56)	11.9 (44)	18.1 (88.5)	17.1 (49)	17.7 (72)	15.9 (55)	16.2 (209)	13.6 (46)	16.5 (95.5)	**28 (468.5)**
ARRDC4<br>MGG00250<br>572aa	13.4 (65.5)	13.4 (65.5)	15.3 (80)	13.8 (36)	15.3 (95.5)	9 (20.5)	**24.7 (631.5)**	14.5 (51)	14.3 (109.5)	19.2 (111)
ARRDC5<br>MGG01045<br>627aa	13 (125)	13.1 (118.5)	**37.3 (929.5)**	18.4 (437.5)	16.6 (140)	10.1 (116.5)	15.9 (145.5)	14 (58)	12.9 (36.5)	16.4 (91)
ARRDC6<br>MGG08765<br>958aa	10.2 (59)	**35.1 (1293.5)**	15.4 (209.5)	8.5 (97.5)	14.1 (89)	7.7 (61)	15 (106.5)	9.2 (40)	7.5 (75)	12.1 (56)
MoPalF<br>MGG01615<br>824aa	11.4 (84)	17.8 (149.5)	16.6 (106)	8.5 (51.5)	**36.9 (1333.5)**	6.6 (19)	16.4 (131)	10 (54)	10.4 (64)	11.9 (73)

Table S3. Primers used in this study.

Primer name	Sequence data
3241u1	AACGATGCCGATCCGAATG
3241u2	AAAATAGGCATTCATTGTTGACCTCCACTACTTGAGAAGCGACCGATGG
3241d1	TCGTCCGAGGGCAAAGGAATAGAGTAGATGTGCTGCTCAGTGTCTTCTCA
3241d2	GGCACATAATCGCAATGGTAAT
Hphm1	TAGTGGAGGTCAACAATGAATG
Hphm2	CATCTACTCTATTCCTTTGCCC
3241n1	CCGGAATTCATGAACAGAAGCCAGAACAGAA
3241n2	CGCGGATCCGGTAGGTAGGTAGGTAGGTAGG
3241ck1	ACGATTATAGCGACAACGAACA
3241ck2	GGACTGGCAAGGATTCAAGAG
Hphck1	TAGTGGAGGTCAACAATGAATG
Hphck2	CATCTACTCTATTCCTTTGCCC
1300-g418u	TCCTCTAGAGTCGACCTGCAGGGAGGTCAACACATCAATGC
1300-g418d	ACGACGGCCAGTGCCAAGCTTTCAGAAGAACTCGTCAAGAAG
3241c1	TATGGAGAAACTCGAGGAACGATGCCGATCCGAATG
3241c2	TACCGAGCTCGAATTCAGCCTTAACACCACGCCATA
3241sb1	AACGATGCCGATCCGAATG
3241sb2	CTTGAGAAGCGACCGATGG
Acr1-q1	AGCCAGCCGACAGACCAT
Acr1-q2	ACCCAAACGAGACGCAAA
Cca1-q1	GGCACTGAAGTTGAAGAGGG

Cca1-q2	CACACCTTTTTGAGCCGAAT
Con7-q1	TCGCAGTCAGATACCTCGTC
Con7-q2	GCCACTATGATTGCTAGCCG
Com1-q1	GAGGACCGGAAGCTCGAATA
Com-q2	TGCCCGAAGTTTGTACGTTG
Cos1-q1	CCGAATCACAGGAGCACATG
Cos1-q2	CGAGGCATGGTAGGTCATCT
Tubulin-q1	ACAACTTCGTCTTCGGTCAG
Tubulin-q2	GTGATCTGGAAACCCTGGAG
arrdc1-xba1	GCTGTACAAGTCTAGAATGGCTGCCTCAGTGAGGG
arrdc1-xba2	GCAGGTCGACTCTAGATTCAATCAAAGGCTTCTTCTGC
rab7-xba1	CACCGGCGGCTCTAGAATGTCGTCCAGAAAGAAGGTTCT
rab7-xba2	GCAGGTCGACTCTAGATTAGCAGGCGCATCCATC
rp27-1	AATCACTAGTGAATTCATAAATGTAGGTATTACCTGTACATTTTAT
rp27-2	TCCCGGGGATGGATCCTTTGAAGATTGGGTTCCTACGA
